# Electromagnetic Interference Shielding Effectiveness of Pure SiC–Ti_3_SiC_2_ Composites Fabricated by Reactive Melt Infiltration

**DOI:** 10.3390/ma18010157

**Published:** 2025-01-02

**Authors:** Mingjun Zhang, Zhijun Ma, Xueqin Pan, Yun Li, Nanlong Zhang, Jiaxiang Xue, Jianfeng Yang, Bo Wang

**Affiliations:** 1 State Key Laboratory for Mechanical Behavior of Materials, Xi’an Jiaotong University, Xi’an 710049, China; zhangmingjun2022@163.com (M.Z.); 17797153065@163.com (Z.M.); 3124102115@stu.xjtu.edu.cn (X.P.); zhangnanlong@stu.xjtu.edu.cn (N.Z.); yang155@mail.xjtu.edu.cn (J.Y.); 2 China Nuclear Power Technology Research Institute Co., Ltd., Shenzhen 518026, China; li_yun1927@163.com

**Keywords:** SiC–Ti_3_SiC_2_, reactive melt infiltration, electrical conductivity, electromagnetic interference shielding effectiveness

## Abstract

Silicon carbide-based titanium silicon carbide (SiC–Ti_3_SiC_2_) composites with low free alloy content and varying Ti_3_SiC_2_ contents are fabricated by two-step reactive melt infiltration (RMI) thorough complete reactions between carbon and TiSi_2_ alloy in SiC-C preforms obtained. The densities of SiC-C preform are tailored by the carbon morphology and volumetric shrinkage of slurry during the gel-casting process, and pure composites with variable Ti_3_SiC_2_ volume contents are successfully fabricated with different carbon contents of the preforms. Due to the increased Ti_3_SiC_2_ content in the obtained composites, both electrical conductivity and electromagnetic interference (EMI) shielding effectiveness improved progressively, while skin depth exhibited decreased consistently. The improvement in the EMI shielding effectiveness of the composite is due to the free electrons being bound to move in the conductive network formed by the Ti_3_SiC_2_ phase, converting electrical energy into thermal energy and reducing the energy of electromagnetic waves. Notably, at a Ti_3_SiC_2_ content of 31 vol.%, the EMI shielding effectiveness of the SiC–Ti_3_SiC_2_ composites in the X-band reached an impressive 62.1 dB, confirming that SiC–Ti_3_SiC_2_ composites can be treated as high-performance EMI shielding materials with extensive application prospects.

## 1. Introduction

The accelerated advancement of 5G technologies and various information systems has resulted in a heightened proliferation of electromagnetic waves across diverse environments, thereby raising significant concerns regarding the disruption of electronic devices and the potential health risks associated with electromagnetic radiation exposure [1,2,3,4]. Consequently, electromagnetic interference (EMI) shielding materials have garnered considerable attention due to their efficiency in attenuating interference and radiation [4].

Silicon carbide (SiC), recognized as a prominent wide-bandgap semiconductor with substantial application potential, is nevertheless encumbered by its limited electrical conductivity. This limitation renders SiC less effective as an EMI shielding material, necessitating enhancements to meet the 20 dB commercial standard [5,6]. In contrast, Ti_3_SiC_2_, a distinctive material characterized by a structural composition that includes both metallic and covalent bonds, exhibits superior electrical conductivity and substantial EMI shielding effectiveness. Notably, within the X-band frequency range (8.2 GHz to 12.4 GHz), Ti_3_SiC_2_ demonstrates EMI shielding effectiveness values ranging from 35 to 54 dB [7,8]. This advantageous characteristic positions Ti_3_SiC_2_ as a compelling candidate for composite materials designed to enhance EMI shielding effectiveness.

In response to the limitations inherent in SiC, extensive research efforts have concentrated on the development of SiC–Ti_3_SiC_2_ composites. Various fabrication methodologies have been investigated, including hot press sintering [9], discharge plasma sintering [10], and reactive melt infiltration (RMI) [11,12,13]. Among these techniques, RMI is particularly noteworthy for its ability to produce composites with higher density and superior dimensional precision compared to the alternative methods. Earlier studies on the preparation of SiC–Ti_3_SiC_2_ composites have encountered challenges, specifically regarding the presence of undesirable residual phases such as TiSi_2_ and TiC, which can adversely affect the purity and overall performance of the resulting materials. For example, research conducted by Xiaomeng Fan identified residual phases in composites prepared using liquid silicon infiltration [11]. Conversely, Nanlong Zhang studied the effect of varying carbon black content in the preforms on the microstructure of the composites after infiltration with liquid TiSi_2_ alloy, while maintaining a fixed porosity of 36% by die pressing [12]. As the carbon content increases, the residual TiSi_2_ content in the composites decreases gradually. When the carbon content in the preform reaches 40 vol.%, the residual TiSi_2_ content in the composites is reduced to only 2 vol.%, and an almost pure SiC–Ti_3_SiC_2_ composite is obtained. This innovative strategy yielded SiC–Ti_3_SiC_2_ composites with a reduced concentration of TiSi_2_ alloy, suggesting a viable pathway for enhancing the fabrication techniques of these composites. However, Zhang has not been able to maintain a high purity while changing the Ti_3_SiC_2_ contents of SiC–Ti_3_SiC_2_ composites.

In this study, pure SiC–Ti_3_SiC_2_ composites with variable Ti_3_SiC_2_ contents are fabricated by gel casting through RF curing and RMI with TiSi_2_ alloy infiltration based on Zhang’s process, to investigate the effect of Ti_3_SiC_2_ contents on EMI shielding effectiveness. The Ti_3_SiC_2_ contents are governed by the carbon contents in the SiC-C preform used for the RMI process, and their appropriate densities are requisite for the decrease in the free TiSi_2_ alloy or free carbon remaining in the composites. A combination of fine nano carbon black and coarse petroleum coke is used to obtain the desired densities by tailoring the volumetric shrinkage during the gel casting, curing, and carbonization. The conductivity and EMI shielding effectiveness of SiC–Ti_3_SiC_2_ composites are systematically investigated.

## 2. Experimental Procedure

### 2.1. Preparation of SiC–Ti_3_SiC_2_ Composites

2130 phenolic resin (PF, Yuyao Meisheng Plasticization Co., Ltd., Yuyao, China), ethylene glycol (EG, Tianjin Damao Chemical Reagent Factory Co., Ltd., Tianjin, China), and powders of α-SiC (10 µm, >99% purity, Shanghai Chaowei Nanotechnology Co., Ltd., Shanghai, China), nano-sized carbon black (CB, N330, 99.7% purity, Tianjin Tianyishiji Co., Ltd., Tianjin, China) and petroleum coke (PC, 20 µm, >99% purity, Tianjin Zhuobang Electronics Co., Ltd., Tianjin, China) are used to prepare the gel-casting slurries and preforms. TiSi_2_ (1~3 mm, 99.5% purity, Jiacheng Rare Metal Materials Co., Ltd., Baoji, China) alloy is used as infiltration and reaction metals in the reactive sintering process.

The preparation of SiC–Ti_3_SiC_2_ composites includes the formation of preforms and RMI. After the raw materials are evenly dispersed, the preforms are obtained through gel casting, curing, and carbonization, in turn [14]. Then, TiSi_2_ alloy is used as infiltrating molten of the preforms for RMI, resulting in the SiC–Ti_3_SiC_2_ composites [12]. The specific steps are as follows:

PF and EG are premixed at first, and then SiC, CB, and PC powders, as well as polyethylene glycol according to 1 wt.% of the SiC as dispersant, are added to the premixture. After mechanical stirring at the rate of 500 r/min for 3 h, benzene sulfonyl chloride as the curing agent is added and the slurries are stirred at the rate of 500 r/min for 0.5 h. Final slurries are obtained after vacuum defoaming for 1 h, and they are purred into a mold. The samples are firstly heated to 80 °C for 2 h for pre-curing treatment, after which they are escalated to 150 °C at the rate of 0.5 °C·min^−1^ and soaked for 16 h. In the process of curing, t the samples are carbonized in a horizontal tube furnace (GSL-1400X, Hefei Kejing Material Technology Co., Ltd., Hefei, China) under argon atmosphere at 800 °C for 4 h with a rapid heating rate of 1.5 °C min^−1^ from room temperature to 600 °C and a slower heating rate of 1 °C min^−1^ from 600 °C to 800 °C, resulting in SiC-C preforms. The TiSi_2_ alloy particles are laid evenly on the graphite paper and the preforms are placed on the alloy. The RMI process is carried out in a vacuum sintering furnace (High Multi-5000, Fujidempa Co. Ltd., Osaka, Japan), with a heating rate of 10 °C·min^−1^ from room temperature to 1450 °C and soaking for 15 min, and rapid heating to 1650 °C at the heating rate of 25 °C·min^−1^ and soaking for 1 h. During the whole process, the vacuum degree does not exceed 20 Pa.

### 2.2. Characterizations

The bulk densities of the preforms and SiC–Ti_3_SiC_2_ composites are measured by the Archimedes method. The phase composition of the composites is characterized by X-ray diffraction (XRD, X Pert PRO, Almelo, The Netherlands) with Cu Kα employed as radiation source and a test range of 20° to 80°. Microstructural analyses of SiC–Ti_3_SiC_2_ composites are conducted with a backscattering electron microscope (BSE, Phenom Pro X, Phenom Scientific Co., Ltd., Cleveland, OH, USA) equipped with energy spectrum analysis equipment employed to characterize the microstructure of SiC–Ti_3_SiC_2_ composites and the chemical elemental composition of the composites. The electrical conductivity of the composites is measured by a resistivity tester (ST2253, Suzhou Jingge Electronic Co., Ltd., Suzhou, China) using the four-point probe method. The S-parameters (S_11_ and S_21_) of the composite material are tested by the waveguide method of the vector network analyzer (VNA, MS4644A, Anritsu, Atsugi, Japan) in X-band (sample size 22.9 mm × 10.2 mm × 2.0 mm). The reflection coefficient (R) and the transmission coefficient (T) are calculated using the S-parameters according to the following equations:

R = |S_11_|^2^ (1)

T = |S_21_|^2^ (2)

Then, the reflection loss (SE_R_), absorption loss (SE_A_), and total shielding effectiveness (SE_T_) can be calculated by the following equations:

SE_R_ = −10 × log (1 − R) (3)

(4) $${\mathrm{SE}}_{A}=-10\;\times \;\log\;(\frac{T}{1-R})$$

SE_T_ = SE_R_ + SE_A_ + SE_M_ (5)

Typically, when the SE_A_ exceeds 10 dB, and in the presence of an electric field and a plane wave, the influence of multiple reflections (SE_M_) can be considered negligible. So,

SE_T_ = SE_R_ + SE_A_ (6)

## 3. Results and Discussions

Based on previous research, the reaction for the formation of SiC–Ti_3_SiC_2_ composites via RMI can be expressed as follows [12]:

7C_(s)_ + 3TiSi_2(l)_ → 5SiC_(s)_ + Ti_3_SiC_2(s)_ (7)

According to Equation (7), a complete reaction of pure carbon preform will produce 58 vol.% SiC and 42 vol.% Ti_3_SiC_2_, without consideration of the residual TiSi_2_ alloy. Consequently, to obtain SiC–Ti_3_SiC_2_ composites with no free TiSi_2_ alloy remaining, SiC-C preforms are designed to achieve Ti_3_SiC_2_ contents between 20 and 33 vol.% after the RMI process. To investigate the effect of the volume content of Ti_3_SiC_2_ on the electromagnetic shielding effectiveness of SiC–Ti_3_SiC_2_ composites, SiC–Ti_3_SiC_2_ composites with Ti_3_SiC_2_ contents of 20 vol.%, 24 vol.%, and 33 vol.% are designed and prepared. As the Ti_3_SiC_2_ content in the SiC–Ti_3_SiC_2_ composites after RMI increases, the corresponding carbon content in the preforms for SiC–Ti_3_SiC_2_ composites also increases, while the space (pores) is also required to increase to accommodate the reaction products as SiC and Ti_3_SiC_2_ within the preforms.

As the porosities directly influence the extent of the TiSi_2_ alloy infiltration, high or low porosity can result in the presence of residual TiSi_2_ or carbon. In order to prepare SiC–Ti_3_SiC_2_ composites with minimal residual TiSi_2_ content, precise control of the bulk density of the preforms is essential. According to the reaction (7), the volume of solid phase reaction products (SiC, Ti_3_SiC_2_) is 2.273 times that of solid phase reactant (C). Based on this reaction and the corresponding volume change in the solid phase, the relation between the theoretical porosity (P) of the preforms without residual TiSi_2_ phase and the volume fraction (V_C_) of C in the preforms can be expressed as follows:

(8) $$P=\frac{1.273V_{C}}{1+1.273V_{C}}$$

Table 1 presents the phase compositions, densities, and porosities of the SiC–C preforms necessary for a complete reaction of SiC/Ti_3_SiC_2_ composites without residual TiSi_2_ alloy corresponding to the different Ti_3_SiC_2_ contents. As illustrated in Table 1, increasing the Ti_3_SiC_2_ contents in the composites necessitates higher porosities of the SiC-C preforms with corresponding carbon contents, because of the greater volume increase occurred during the RMI process. The increase in the content of the conductive phase (Ti_3_SiC_2_) can improve the electrical conductivity of the composites and increase the energy loss of electromagnetic waves entering the composites, thus improving the EMI shielding effectiveness of SiC–Ti_3_SiC_2_ composites.

In this study, the gel-casting method is employed, with the preform volume primarily determined by the contents of EG and PF as the liquid phase. During the conversion from slurry to the solid preform, the volatilization of EG leads to the formation of voids, while PF undergoes polycondensation to produce a three-dimensional network structure, which is subsequently carbonized into activated carbon with a mass loss of approximately 40 wt.%. The volatilization of EG and the conversion process of PF are typically accompanied by a volumetric shrinkage of around 24% during the curing and carbonization stages, as determined from previous experience.

**Figure 1:**
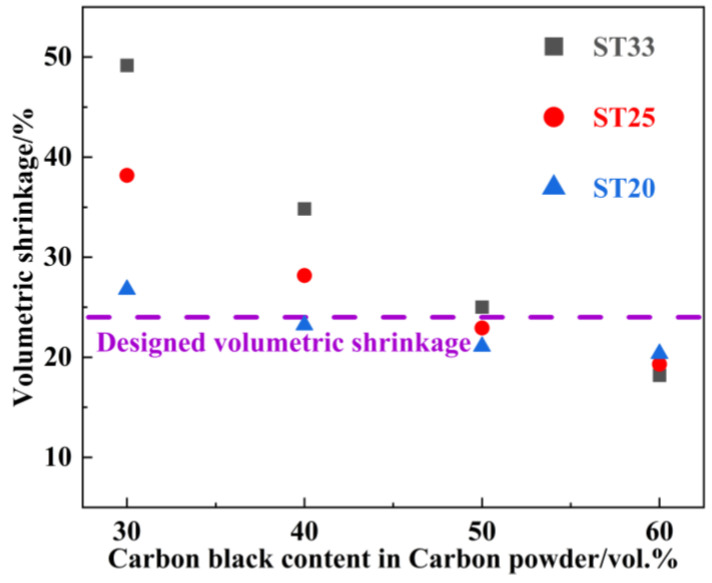
Change of volumetric shrinkage of the SiC-C preforms obtained as in Table 2 with different CB contents in the total carbon powder in the slurries.

Based on the phase compositions of preforms presented in Table 1, the proportions of carbon and residual carbon of PF and SiC are determined, and taking into the consideration the 24% volumetric shrinkage, varying amounts of EG can be defined according to the densities in Table 1, ultimately leading to the slurry formulations of different SiC-C preforms for the SiC–Ti_3_SiC_2_ composites without residual TiSi_2_ alloy in Table 2. Furthermore, the slurry viscosity can be adjusted by modifying the CB content in the carbon source in Table 2 to obtain a desirable volumetric shrinkage of 24%. As illustrated in Figure 1, the volumetric shrinkage of the preforms for the SiC–Ti_3_SiC_2_ composites decreases with the increasing CB content in the carbon powder. When the CB content reaches 40 vol.%, the volumetric shrinkage of the preform for ST20 closely approaches the designed value of 24%. With a CB content of 50 vol.%, the volumetric shrinkage of preforms for ST25 and ST33 also have a similar corresponding relationship with the designed value of 24%. This phenomenon can be attributed to the fact that the smaller-sized CB particle facilitates particle stocking, and enhances resistance to volumetric shrinkage in curing and carbonation. Consequently, the CB content of 40 vol.% is used for the preparation of the preform for ST20 and the CB content of 50 vol.% is used for the preparation of preforms for ST25 and ST33. The corresponding volumetric shrinkage for the preforms are 22.9%, 23.2%, and 25.1%, which align well with the designed value for Table 2.

The densities of SiC-C preforms and SiC–Ti_3_SiC_2_ composites are measured and compared with theoretical values to evaluate the degree of reaction within the preforms. The measurement results are summarized in Table 3. The resulting densities being similar to the theoretical values signifies that complete reaction occurred, facilitating high densification of the samples.

**Figure 2:**
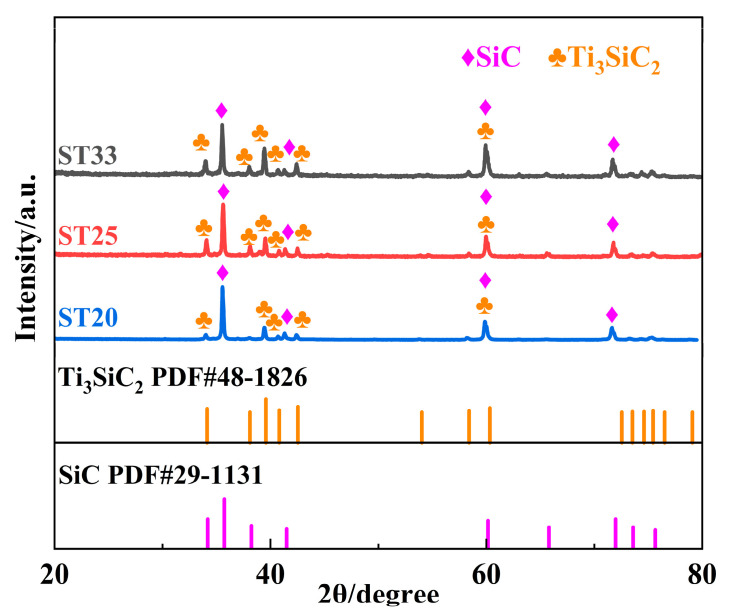
XRD pattern for obtained SiC–Ti_3_SiC_2_ composites.

Figure 2 presents the X-ray diffraction spectra of the obtained SiC–Ti_3_SiC_2_ composites. The composites exhibit similar phase compositions, with distinct diffraction peaks corresponding to the SiC and Ti_3_SiC_2_ phases; notably, no peaks indicative of the TiSi_2_ phase are observed. The relative enhancement of the main peak associated with the Ti_3_SiC_2_ phase is attributed to the increased carbon content in the preforms, which is accompanied by a decrease in the intensity of the SiC peak.

**Figure 3:**
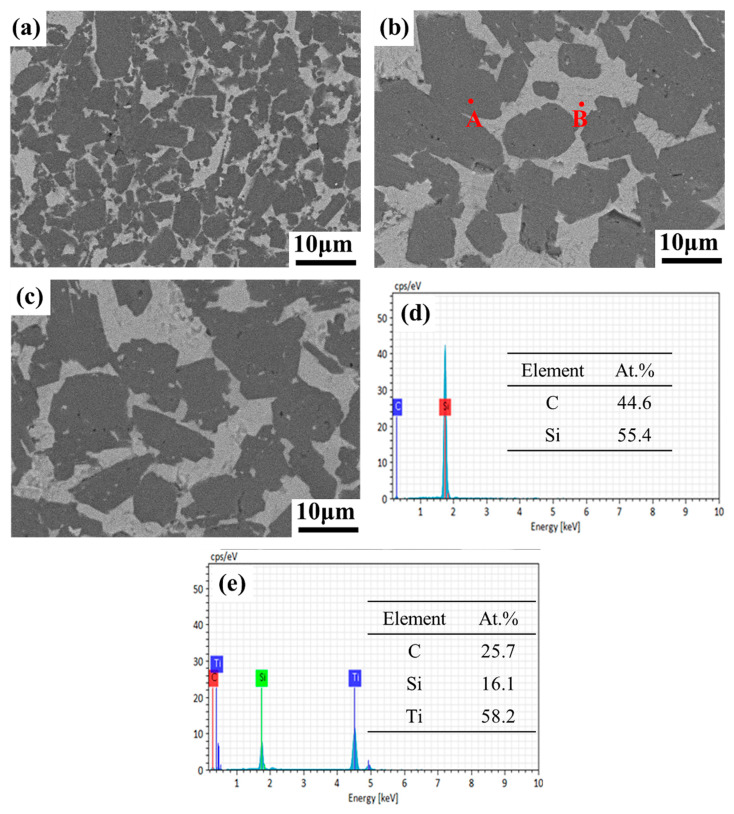
BSE images of polished surface of SiC–Ti_3_SiC_2_ composites (**a**) ST20, (**b**) ST25, (**c**) ST33; (**d**,**e**) EDS analysis at points A, B for sample ST25.

Figure 3 presents a backscattered electron (BSE) image of the polished surfaces of the composites. In these images, lighter gray regions correspond to the Ti_3_SiC_2_ phase, while darker regions represent the SiC phase. The significant differences in atomic numbers between the two phases facilitate their clear distinction [11]. Each phase is uniformly distributed across the composites, with the Ti_3_SiC_2_ phase notably located at the grain boundaries of the SiC phase, and no evidence of the TiSi_2_ phase is present. Furthermore, the BSE images clearly reveal that as the Ti_3_SiC_2_ content increases, progressively developing a network structure. And as illustrated in Figure 3d,e, there is a strong correlation between the atomic percentages of Si, Ti, and C at points A and B, matching the atomic percentages within the respective SiC and Ti_3_SiC_2_ phases.

In fact, the reaction between C and infiltrating TiSi_2_ alloy occurs according to two distinct reactions [12]:

3C_(s)_ + TiSi_2(l)_ → 2SiC_(s)_ + TiC_(s)_ (9)

7TiC_(s)_ + 2TiSi_2(l)_ → SiC_(s)_ + 3Ti_3_SiC_2(s)_ (10)

During the reactive sintering process, the molten TiSi_2_ alloy infiltrates the preform through capillary force, and firstly reacts with C to produce SiC and TiC, according to Equation (9). Table 4 shows the calculated phase composition in the preforms after the first reaction (Equation (9)) is completed. It is obvious that the contents of the TiSi_2_ alloy in the materials after the first reaction are in range of 7–14 vol.%, indicating a good infiltration channel. Next, the TiC formed in this initial reaction subsequently reacts with the residual molten TiSi_2_ alloy to form additional SiC and Ti_3_SiC_2_, as illustrated in reaction Equation (10).

The theoretical contents of the Ti_3_SiC_2_, SiC, and TiSi_2_ phases can also be clearly calculated based on the densities of the preforms. To achieve precise quantification of the phase contents within the composites, pixel statistical analysis is performed and compared with the theoretical values, as shown in Table 5. Significantly, the actual phase contents of the three composites correlate well with theoretical predictions, and the TiSi_2_ phase is almost not detectable in any of the composites. These findings demonstrate that the densities of the preforms are effectively controlled, leading to the formation of high-purity SiC–Ti_3_SiC_2_ composites following RMI.

**Figure 4:**
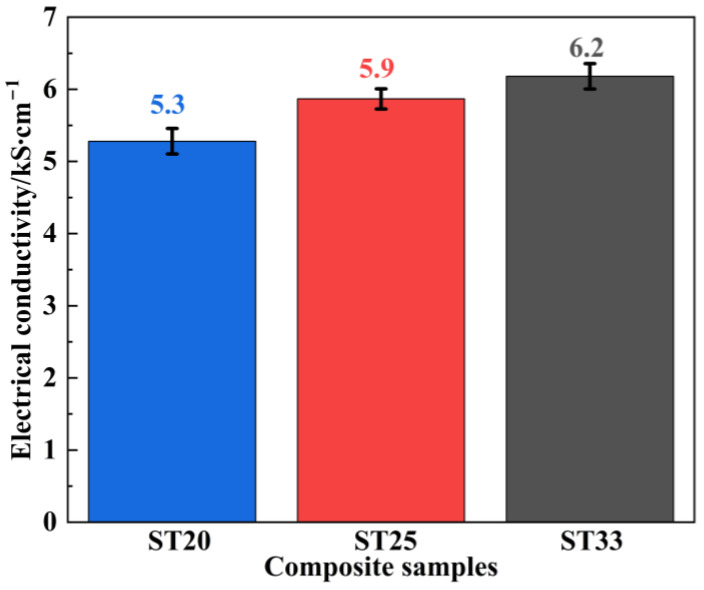
Electrical conductivity of the SiC–Ti_3_SiC_2_ composites with different Ti_3_SiC_2_ contents.

Figure 4 illustrates the electrical conductivity of the SiC–Ti_3_SiC_2_ composites. With the increase in the Ti_3_SiC_2_ contents, the electrical conductivity is increased continuously, and conductivities of 5.3 kS/cm, 5.9 kS/cm, and 6.2 kS/cm are obtained for the samples of ST20, ST25, and ST33, respectively. Although the conductivities of the SiC–Ti_3_SiC_2_ composites are relatively weaker compared to metal-based shielding materials such as Al (40 kS/cm) [15], compared with carbon materials used for EMI shielding, such as reduced graphene oxide composites (1.1 kS/cm) [16], due to the good conductivity of the Ti_3_SiC_2_ phase (45 × 10^3^ kS/cm) [11] and the high purity of the obtained SiC–Ti_3_SiC_2_ composites, the formation of the conductive network is ensured, and the conductivities of the SiC–Ti_3_SiC_2_ composites are significantly improved and suitable for EMI shielding in the X-band. As a typical semiconductor, the primary SiC phase within the composite materials does not significantly enhance conductivity [17]. The variations in the conductivity among the composites can be attributed to differences in the content and distribution of the Ti_3_SiC_2_ phase. As the volume fraction of the highly conductive phase Ti_3_SiC_2_ ascends in ST20, ST25, and ST33 composites (18.2 vol.%, 25.0 vol.% and 31.0 vol.%, respectively), the conductive network is further improved, facilitating electron mobility and enhancing the overall conductivity of the composites.

**Figure 5:**
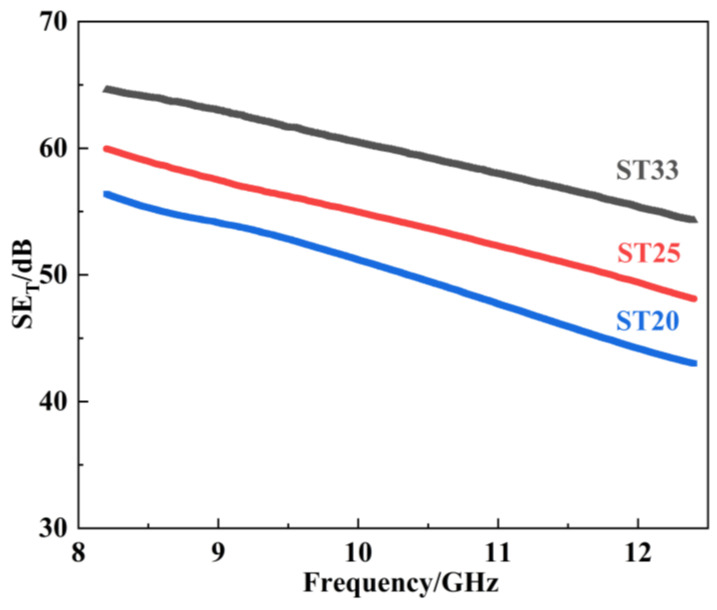
Variation of SE_T_ as a function of frequency for the SiC–Ti_3_SiC_2_ composites with different Ti_3_SiC_2_ contents in X-band.

The evaluation of the EMI shielding performance of materials is commonly quantified by the EMI shielding effectiveness [18,19]. Figure 5 illustrates SE_T_ for SiC–Ti_3_SiC_2_ composites in X-band with varying amounts of Ti_3_SiC_2_ content. All the composites exhibit SE_T_ values exceeding 30 dB, demonstrating exceptional EMI shielding effectiveness capable of attenuating over 99% of the electromagnetic wave, significantly surpassing the prevailing commercial standard of 20 dB. Notably, the SE_T_ is significantly influenced by the Ti_3_SiC_2_ content; as the volume fraction of Ti_3_SiC_2_ increases from 18.2 vol.% to 31.0 vol.%, the average SE_T_ improves from 46.8 dB to 62.1 dB.

**Figure 6:**
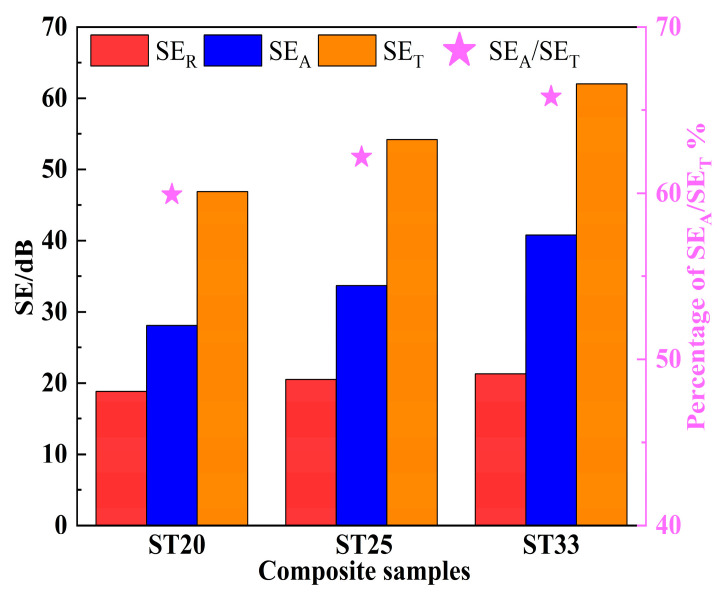
Average SE_T_, SE_A_, SE_R,_ and SE_A_/SE_T_ of SiC–Ti_3_SiC_2_ composites with different Ti_3_SiC_2_ contents.

The contribution of both SE_A_ and SE_R_ to the overall SE_T_ are illustrated in Figure 6, and the improvement in the SE_T_ is mainly due to the contribution of the SE_A_. As the Ti_3_SiC_2_ content increases, the average SE_R_ demonstrates a modest enhancement from 18.7 dB to 21.3 dB. In contrast, the average SE_A_ demonstrates a more substantial increase from 28.1 dB to 40.8 dB. Furthermore, the ratio of SE_A_ to SE_T_ also shows an upward trend with a higher Ti_3_SiC_2_ content. The SE_A_ represents the absorption loss for electromagnetic waves entering the interior of the composites. When electromagnetic waves penetrate the material, they induce currents within the conductive Ti_3_SiC_2_ phase, causing free electrons to move and convert electrical energy into thermal energy. Hence, a higher Ti_3_SiC_2_ content correlates with stronger attenuation of electromagnetic waves. These results emphasize the increasing importance of the absorption loss in enhancing the EMI shielding effectiveness of SiC–Ti_3_SiC_2_ composites.

**Figure 7:**
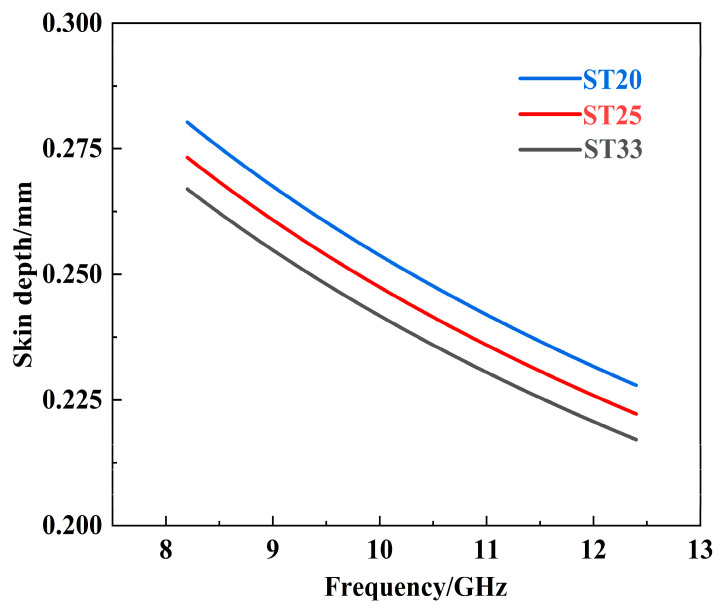
Skin depth of the SiC–Ti_3_SiC_2_ composites with different Ti_3_SiC_2_ contents as a function of frequency in X-band.

To further investigate the EMI shielding effectiveness, a comprehensive comparison of the composites with varying Ti_3_SiC_2_ contents is conducted through skin depth analysis. Skin depth quantifies how deeply an electromagnetic wave penetrates a material before its amplitude attenuates, decreasing to 1/e (where e ≈ 2.718) of its original value [17]. It can be calculated using the following formula:

(11) $$\delta =\frac{1}{\sqrt{\pi f\mu \sigma }}$$

where δ represents the skin depth, f denotes the frequency of the electromagnetic wave, and σ stands for the conductivity of the composite material. Additionally, the permeability μ is expressed as μ = μ_0_ × μ_r,_ where μ_0_ = 8.854 × 10^−7^ and μ_r_ = 1. As illustrated in Figure 7, the skin depth of composites ST20, ST25, and ST33 exhibit a gradual decrease with increasing Ti_3_SiC_2_ content. This reduction in skin depth indicates a shorter propagation distance for electromagnetic waves within the composite material, thereby confirming the improvement in absorption loss of SiC–Ti_3_SiC_2_ composites against electromagnetic waves. This phenomenon is consistent with the significant enhancement of SE_A_ observed with increased Ti_3_SiC_2_ content. It is further confirmed that the enhancement of absorption loss in the composites is directly corelated to the increased content of Ti_3_SiC_2_, which promotes the interaction between the composite materials and the electromagnetic waves, ultimately improving the EMI shielding effectiveness.

**Figure 8:**
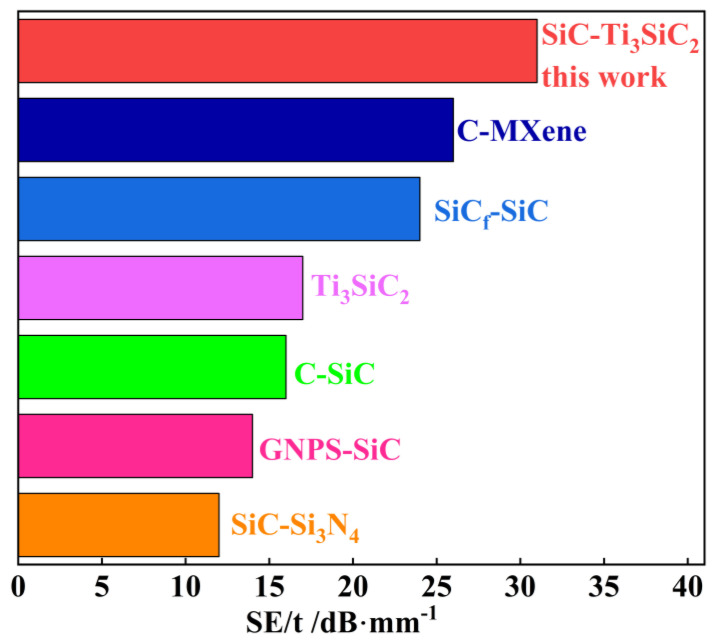
Comparison of EMI shielding effectiveness of various EMI shielding materials(Ti_3_SiC_2_ [4], C-Mxene [20], SiC_f_-SiC [21], C-SiC [22], GNPS-SiC [23] and SiC-Si_3_N_4_ [24]) reported in the literature.

Figure 8 compares the shielding capability of various EMI shielding materials reported in the literature [4,20,21,22,23,24]. The thickness of different materials in the experiment is different; in order to more accurately evaluate the EMI shielding effectiveness of the materials, a more realistic parameter is to divide SE by the material thickness (SE/t) [23]. As can be seen from Figure 8, the SiC–Ti_3_SiC_2_ composites in this study shows the highest EMI shielding efficiency (the attenuation per unit thickness of SiC–Ti_3_SiC_2_ composite with 33 vol.% Ti_3_SiC_2_ is calculated to be around 31 dB/mm) in all EMI materials reported in the literature. The superior effectiveness of SiC–Ti_3_SiC_2_ composites for shielding electromagnetic waves is also demonstrated.

## 4. Conclusions

In this study, pure SiC–Ti_3_SiC_2_ composites are successfully fabricated through RMI in gel-casted C-SiC preforms with a certain density thorough a complete reaction between carbon and TiSi_2_ alloy. The composites with different Ti_3_SiC_2_ contents and free of TiSi_2_ are obtained by varied carbon contents and the determined preform density by tailoring the carbon morphology and relative contents, respectively. Composites with respective Ti_3_SiC_2_ contents of 18.2 vol.%, 25.0 vol.%, and 31.0 vol.% are fabricated. With the increase in Ti_3_SiC_2_ content, the electrical conductivity rises from 5.3 kS/cm to 6.2 kS/cm, and the EMI shielding effectiveness increases from 46.8 dB to 62.1 dB. This enhancement is attributed to the improvement in the absorption loss with the conductive network formation by the Ti_3_SiC_2_ phase, which restricts the movement of free electrons and increases the attenuation of electromagnetic waves. Furthermore, the reduction in skin depth with the increasing Ti_3_SiC_2_ contents indicates a decrease in the propagation distance of electromagnetic waves within the material, further demonstrating the superior EMI shielding effectiveness of the obtained composites. All the results confirm that the Ti_3_SiC_2_ phase is an effective EMI shielding additive, and the SiC–Ti_3_SiC_2_ composites can be treated as high-performance EMI shielding materials with extensive application prospects.

## Figures and Tables

**Table 1:** Phase composition, densities, and porosities of the SiC-C preforms as well as corresponding Ti_3_SiC_2_ contents for SiC–Ti_3_SiC_2_ composites after complete reaction without residual TiSi_2_ alloy.

ST Composites	Phase Compositions of Preforms/vol.%		Densities of Preforms/g·cm^−3^	Porosity of Preforms/%	Ti_3_SiC_2_ Content of Composites/vol.%
	SiC	C			
ST20	60	40	1.73	34	20
ST25	40	60	1.32	43	25
ST33	20	80	1.04	51	33

**Table 2:** Slurry compositions of different SiC-C preforms for SiC–Ti_3_SiC_2_ composites with the consideration of 24% volumetric shrinkage and no free TiSi_2_ alloy.

ST Composites	Slurry Compositions of the SiC-C Preforms for Composites/vol.%			
	Carbon Powder	SiC Powder	Ethylene Glycol	Phenolic Resin
ST20	10.7	31.2	30.6	27.5
ST25	19.1	19.3	34.6	27.0
ST33	21.8	7.9	43.9	26.4

**Table 3:** Theoretical densities of preforms and composites after complete reaction without residual TiSi_2_ alloy and the actual density of the obtained preforms and composites.

ST Composites	The Theoretical Density/g·cm^−3^		The Actual Density/g·cm^−3^	
	Preforms	Composites	Preforms	Composites
ST20	1.73	3.47	1.73	3.44
ST25	1.32	3.54	1.33	3.51
ST33	1.04	3.64	1.05	3.62

**Table 4:** The phase compositions of the preforms for SiC–Ti_3_SiC_2_ composites after the completion of the first reaction obtained by calculation based on Equation (9).

ST Composites	Phase Compositions of the Preforms after the First Reaction/vol.%		
	SiC	TiC	TiSi_2_
ST20	80.5	12.1	7.4
ST25	71.8	17.5	10.7
ST33	63.7	22.5	13.8

**Table 5:** The Ti_3_SiC_2_, SiC, and TiSi_2_ phase contents of SiC–Ti_3_SiC_2_ composites obtained by calculation based on perform densities and pixel statistics on SEM micrographs.

ST Composites	The Volume Fraction of Each Phase by Calculation/vol.%			The Volume Fraction of Each Phase by Pixel Statistics/vol.%	
	Ti_3_SiC_2_	SiC	TiSi_2_	Ti_3_SiC_2_	SiC
ST20	21.0	78.2	0.8	18.2	81.8
ST25	25.7	73.4	0.9	25.0	75.0
ST33	33.2	65.8	1.0	31.0	69.0

## Data Availability

The original contributions presented in this study are included in the article. Further inquiries can be directed to the corresponding authors.
